# Multimodal imaging of mixed epithelial and stromal tumor of the kidney: Case series

**DOI:** 10.1097/MD.0000000000040656

**Published:** 2024-11-29

**Authors:** Ning Wang, Li Zhang, Shuo Wang, Xiang-Yang Chang, Bao-Gen Zhao, Yong Wang

**Affiliations:** a Department of Radiological and Nuclear Medicine, The First Hospital of Hebei Medical University, Shijiazhuang, Hebei Province, China; b Department of Medical Imaging, The Second Hospital of Hebei Medical University, Shijiazhuang, Hebei Province, China.

**Keywords:** Bosniak Grade, cystic nephroma, imaging diagnosis, mixed epithelial stromal tumor of the kidney, multimodal imaging

## Abstract

**Background:**

To investigate the clinical features, imaging manifestations, and pathological characteristics of mixed epithelial stromal tumor of the kidney (MESTK) to improve the understanding of MESTK and the accuracy of preoperative imaging diagnosis.

**Methods:**

The clinical, imaging, and pathological data of 3 patients with MESTK confirmed by postoperative pathology were retrospectively analyzed.

**Results:**

Three patients with MESTK were middle-aged women with no obvious clinical symptoms. The main image representation showed a multilocular cystic or solid-cystic mass, with the larger masses protruding from the kidney contour, with multiple separations, which could be of unequal thickness. The cystic component showed no enhancement on enhancement scans, whereas the segregations and solid components showed gradual and obvious enhancement. Microscopic pathology was characterized by the biphasic differentiation of mesenchymal and epithelial components.

**Conclusion:**

MESTK is a rare, mostly benign tumor that appears as a multilocular cystic or cystic solid, with progressive marked enhancement of the septal and solid components on enhanced scans. This imaging feature is helpful for the diagnosis of MESTK.

## 1. Introduction

Mixed epithelial and stromal tumors of the kidney (MESTK) were first reported by Michal and Syrucek in 1998.^[1]^ Because of the pathological characteristics of the lesion, which contains both mesenchymal and epithelial components, this tumor was officially named renal mixed epithelial mesenchymal tumor by the World Health Organization (WHO) classification of renal Tumor Histology and Genetics in 2004. These lesions are rare, and the main imaging findings are multicystic or solid-cystic masses; therefore, this tumor is often classified as Bosniak Grade III–IV preoperatively,^[2]^ which is difficult to diagnose preoperative because of the lack of specificity in clinical and imaging findings, and the diagnosis relies mainly on histopathology and immunohistochemical examination. Therefore, through the analysis of the imaging manifestations of a series of cases, we hope to improve people’s understanding of MESTK, so as to improve the correct diagnosis rate of preoperative imaging.

## 2. Materials and methods

### 2.1. Clinical data

This study was approved by the ethics committee of the Second Hospital of Hebei Medical University (2020-R122). All patients in this study signed informed consent forms. We retrospectively analyzed the clinical, imaging, and pathological data of 3 patients with MESTK confirmed by surgery and pathology from March 2022 to June 2023. The 3 patients with MESTK were female, aged 36 to 56 years, with a mean age of 44 years. Case 1 underwent left mastectomy and axillary lymph node biopsy for breast cancer in May 2020, and found right nephropathy during hospitalization. The patient had no fever or gross hematuria, and was not treated. The patient suffered from hypertension and diabetes, which were well controlled after drug treatment. Other physical examinations revealed no major abnormalities. While the other 2 patients had no obvious symptoms and were accidentally found on physical examination. None of the 3 patients had a history of oral contraceptive use. None of the 3 patients had a family history associated with renal tumors. All 3 patients underwent computed tomography (CT) or magnetic resonance imaging (MRI) plain scans and enhanced examinations before surgery and were diagnosed with Bosniak IIF–IV complex cysts. Two patients underwent laparoscopic partial nephrectomy and 1 underwent radical nephrectomy (Table 1).

**Table 1:** Detailed summary of patients’ data in the research.

Case	Gender	Age (yr)	Medical history	Clinical symptoms	The time from lesion discovery to surgery	Preoperative radiological diagnosis	Operation	Recurrence
1	Female	56	Postoperative of left breast cancer	None	22 mo	Bosniak IV	Partial nephrectomy	No
2	Female	41	None	None	6 mo	Bosniak IIF	Radical nephrectomy	No
3	Female	36	None	None	1 mo	Bosniak III	Partial nephrectomy	No

### 2.2. Examination techniques

SOMATOM Force dual-source CT was used in 2 patients for plain scanning and contrast enhancement scanning. The scanning parameters were as follows: 120 kv, 250 mA, slice thickness of 1.25 mm, the pitch of 1.4, for enhanced CT, iodixanol injection was used at a rate of 3.5 to 4 mL/s (1.5 mL/kg, 80–100 mL). Unenhanced scanning was performed before contrast agent injection, and then the corticomedullary phase (30 seconds), nephrographic phase (70 seconds), and excretion phase (3 to 5 minutes) were obtained. Achieva 3.0T high field magnetic resonance scanner (Philips) was used to perform MRI examinations in 1 patient. The scanning parameters were as follows. T1-weighted imaging (T1WI): repetition time 6.75 ms, echo time 2.39 ms; FsT2WI: repetition time 2000 ms, echo time 75 ms; Fov 380×380 mm; slice thickness 3.0 mm. Dynamic enhanced scanning was performed by injecting the contrast agent dimeglumine gadopentetate (Gd-DTPA) into the forearm vein.

### 2.3. Imaging analysis

The imaging data of 3 patients were analyzed by 2 radiologists who had worked for more than 5 years, and the location, size, shape, capsule wall and separation, edge, and enhancement mode of each lesion were recorded.

## 3. Result

### 3.1. Imaging findings

**Figure 1.**
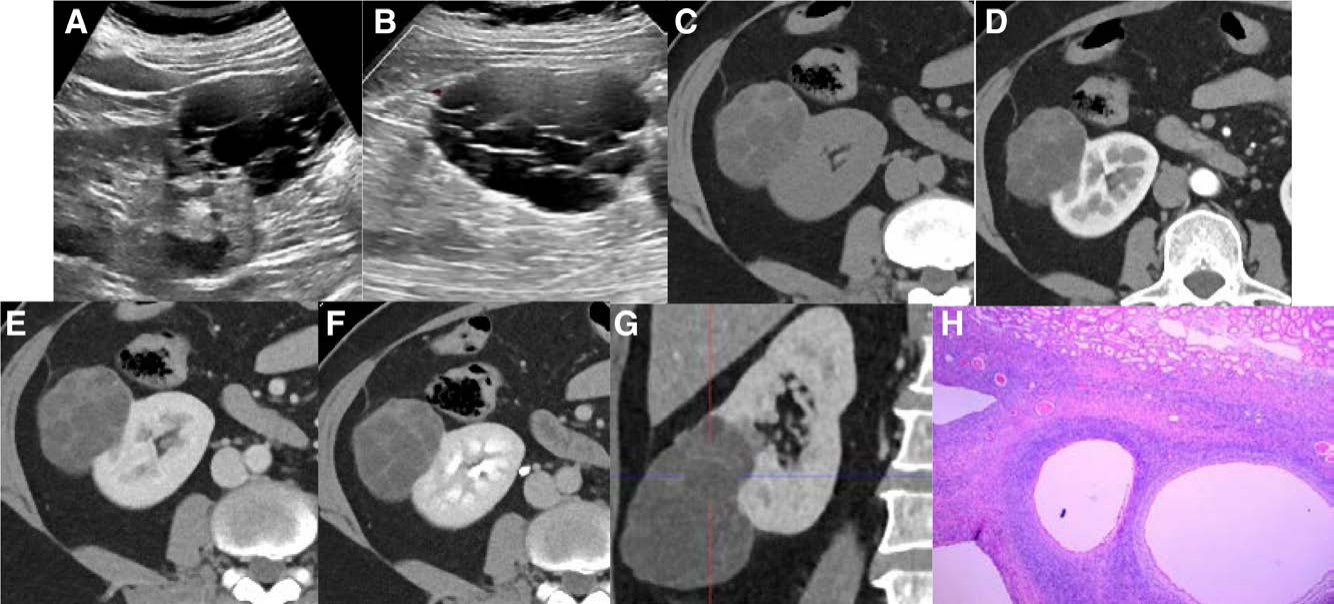
MESTK in a 56-year-old woman. (A) Ultrasound examination revealed cystic lesions in the lower pole of the right kidney (31 months before this study). (B) Ultrasound examination revealed cystic masses in the lower extremity of the right kidney with an irregular shape, most of which protruded from the renal parenchyma and showed uneven separation (20 months before this study). (C–G) A non-contrast and contrast enhanced CT scan showed that the multilocular cystic lesion of the lower right kidney was protruding outside the renal contour without invading the renal pelvis and calyces, and the cyst wall showed uneven thickness. No obvious enhancement was observed in the cystic part, and the separation was mildly enhanced (9 months before this study). (H) Histological findings of MESTK (HE × 40). Microscopically, the lesions were biphasic, with multiple cysts in ovarian stromal spindle cells and a single layer of flat or cubic epithelium covering the cyst wall.

**Figure 2.**
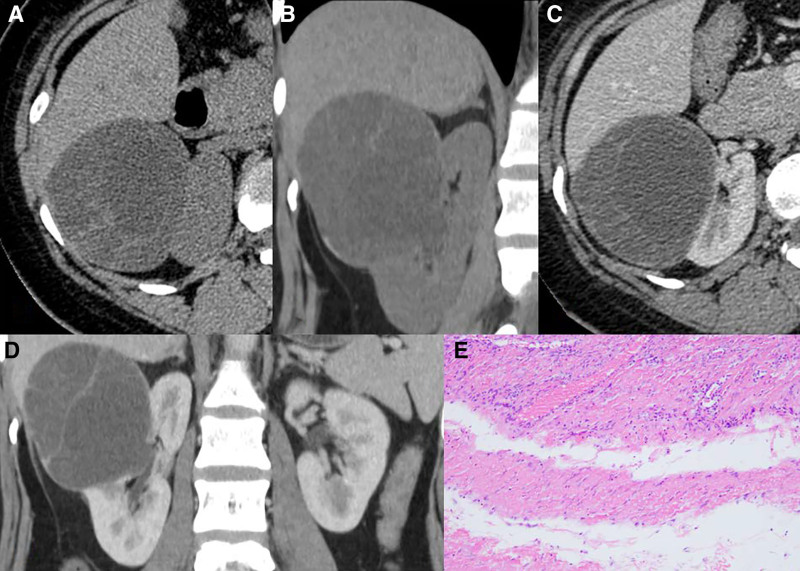
MESTK in a 41-year-old woman. (A, B) A non-contrast CT scan showed multilocular cystic low-density lesions in the right kidney. (C, D) A contrast enhanced CT scan showed that there is no obvious enhancement in the cystic part, and the separation was mildly enhanced. (E) Histological findings of MESTK (HE × 100).

**Figure 3.**
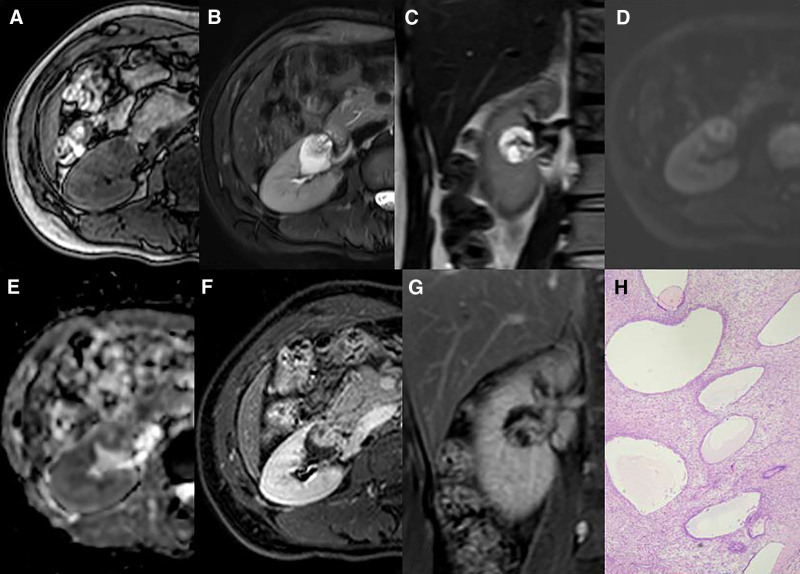
MESTK in a 36-year-old woman. (A) T1WI showed a circular lesion in right kidney with slightly lower signal. (B, C) T2WI showed that the lesion was circular, cystic components showed high signal, solid components and separation showed equal signal. (D, E) DWI showed a slightly higher signal of the solid component of the lesion, and ADC showed an equal or slightly lower signal. (F, G) On enhanced scan, the solid component of the lesion was significantly enhanced, while the cystic component was not. (H) Histological findings of MESTK (HE × 40).

The lesions in all 3 patients were located in the right kidney. The lesions were multilocular cystic or cystic-solid, round or round-like, with clear borders, clearly demarcated from the normal renal parenchyma, and varying in size, with the larger ones often protruding beyond the kidney contour and the smaller ones being located in the kidney contour. Two patients (Case 1 and Case 2) underwent plain scanning and contrast enhancement CT scanning, and showed cystic hypo-dense shadows, with cystic components of approximately 3 to 10 Hounsfield unit, and with multiple separations and uneven thicknesses. Contrast enhancement scanning showed no enhancement of the cystic component of the lesions, but significant strengthening of the separations (Figs. 1 and 2). Plain and contrast enhancement MRI scanning was performed in 1 patient (Case 3), and the lesion was a cystic solid. MRI showed long T1WI and long T2-weighted imaging (T2WI) signals in the cystic components and iso/slightly longer T1WI and iso/or slightly longer T2WI signals in the solid components. The solid components were diffusion-limited on diffusion-weighted imaging (DWI). No obvious enhancement was observed in the cystic components on enhanced scanning, while the solid components showed gradual and obvious enhancement with time delay (Fig. 3, Table 2).

**Table 2:** Imaging characteristics of the cases observed in the research.

Case	Location	Size (cm)	Shape	Boundary	Nature	CT	MRI	Contrast enhancement
1	Right	8 × 6 × 6	Round-like	Clear	Multilocular cystic	Low-density	–	No enhancement of the cystic component of lesions, but significant strengthening of the separations
2	Right	8 × 6 × 5	Round-like	Clear	Multilocular cystic	Low-density	–	No enhancement of the cystic component of lesions, but significant strengthening of the separations
3	Right	2 × 2 × 1	Round-like	Clear	Cystic-solid	–	T1WI: low-signal T2WI: high-signal DWI: solid components were High-signal	No enhancement of the cystic component of lesions, but the solid components showed gradual and obvious enhancement with time delay

CT = computed tomography, DWI = diffusion-weighted imaging, MRI = magnetic resonance imaging, T1WI = T1-weighted imaging, T2WI = T2-weighted imaging.

### 3.2. Pathological and immunohistochemical

The tumors were generally multilocular cystic and showed biphasic differentiation under a microscope. Multiple cysts were observed in the ovarian stromal spindle cells, and the cyst wall was covered with a single flat or cubic epithelium. Immunohistochemistry results showed positive estrogen and progesterone receptor expression in spindle stromal cells, whereas epithelial cells expressed Ckpan/CK7, EMA and PAX-8.Eventually, all 3 tumors were diagnosed as MESTK.

### 3.3. Postoperative follow-up

The 3 patients had no recurrence at 6 to 21 months of postoperative follow-up and this result reduces the psychological stress of patients.

## 4. Discussion

MESTK was first reported by Michal and Syrucek in 1998.^[1]^ MESTK has been referred to as adult mesodermal nephroma, multilocular cystic nephroma with ovarian cell-like stroma, and pyelocystic hamartoma.^[3,4]^ Because of the pathological characteristics of the lesions, which contain both mesenchymal and epithelial components, these lesions were officially classified as renal mixed epithelial mesenchymal tumors according to the 2004 World Health Organization (WHO) classification of renal tumor Histology and Genetics. In the 2016 WHO classification of kidney tumors, adult cystic nephroma (ACN) and mixed epithelial and stromal tumors (MEST) were included in the mixed epithelial mesenchymal tumor family as a tumor lineage because of the similar clinical, morphological, and immunohistochemical manifestations of ACN and MEST. In addition, the mRNA expression profiles of these tumors are similar to those of other kidney tumors.^[5,6]^

MESTK is a rare renal tumor, accounting for 0.2% to 1.6% of all renal tumors,^[2,3,7]^ and several hundred cases have been reported in the literature.^[3,8]^ MESTK is more common in perimenopausal women, with an average age of incidence of 45 to 46 years and a male-to-female ratio of approximately 1:10 to 1:6.^[4,8,9]^ Most patients have a history of estrogen therapy, although there are a few reports of MESTK in young men.^[4,8]^ Most patients have no obvious symptoms or alterations in kidney function, and the tumors are mostly found during a physical examination or routine examination for other causes.^[8]^ Some patients have symptoms such as abdominal pain, palpable abdominal mass, hematuria, or urinary tract infection.^[4,8]^ In this study, the patients were middle-aged female who did not receive hormone therapy and presented with no obvious symptoms before surgery.

Although the origin of MESTK remains unclear, it is believed to be related to residual tissues of Müller’s ducts. The occurrence of this tumor is frequently associated with hormone imbalance or long-term hormone therapy, and is therefore assumed to be related to sex hormones.^[6]^ In terms of histopathology, tumors can be predominantly epithelial or stromal or contain a combination of epithelial and stromal components. In general, most tumors are epithelial type.^[6]^ However, there is a clear tendency for tumors to contain multiple types of epithelial and stromal components.^[10]^ Epithelium is characterized by cystic or tubular structures of varying sizes or complex papilliform structures. Flattened, cubic, and boot-shaped epithelia were the most common forms. Some tumors have densely packed small branched glands, round glands lined with tall cubic epithelium similar to that found in thyroid follicles, and lobular papillae similar to those found in phylloid tumors or in the urothelium. The most common types of stroma are fibrous stroma with few cells and spindle stroma with abundant cells, followed by stroma with smooth muscle differentiation, edematous stroma, ovarian stroma, endometrioid stroma, and adipose tissue.^[11,12]^ Hypocellular fibrous and adipose stroma are more common in large tumors, whereas multicellular stroma is more common in small tumors. Calcification, chronic inflammation, and necrotic components are observed in these lesions.^[5]^ Immunohistochemical analysis has shown that tumors often express various epithelial and stromal components, and MESTK lesions are frequently positive for estrogen and progesterone receptors.^[13]^ High expression levels of estrogen and progesterone receptors are common in the stromal components of female patients, whereas this finding is less common in male patients. Positive expression of androgen receptors is associated with epithelial characteristics of Müller tubulogenesis.^[6]^

Most MESTKs are benign renal tumors that lack specific imaging features. They are mostly multilocular cystic with clear boundaries, solid cystic, or solid. Tumors can be detected using ultrasonography, CT, and MRI, although achieving a definite diagnosis is difficult. Tumors are approximately 2 to 24 cm in size, with an average size of 6 cm, and are rarely > 10 cm.^[14]^ The lesions may have a capsule and are mostly located in the renal parenchyma, frequently protruding towards the renal contour or invading the renal pelvis and proximal ureter.^[15]^ CT and MRI results showed a single cystic-solid renal mass containing different proportions of solid components, a smooth cyst wall, and no obvious wall nodules. The cystic cavity contents consist of cystic fluid, which may be accompanied by bleeding. No enhancement of the cystic cavity was observed. Enhancement of the solid and separation components was significantly lower than that of the renal cortex and showed delayed enhancement. The delayed enhancement may be due to the higher content of collagen fibers in the solid components, which limits the diffusion of contrast agents in the tumor.^[2]^ The solid spindle interstitial component of the lesion was characterized by a slightly higher density, and the corresponding T2WI sequence emitted a low signal. Because of degeneration of the solid component, such as hyaluronic degeneration, edema, or fibrosis, the T2WI sequence can emit a high signal.^[16]^ The solid component showed mild progressive enhancement. According to the ratio of the maximum diameter of the solid component to the maximum diameter of the tumor at the maximum lesion level, some researchers classified tumors as solid type if the ratio was > 60%, or cystic type if the ratio was < 60%. The results showed that the average diameter of cystic lesions was larger than that of solid lesions, which could be attributed to the fact that cystic lesions contain more epithelial components, which have a greater proportion of papillary structures and small glandular components, secreting large amounts of liquid components to form multiple cystic cavities of varying sizes.^[2]^ Renal sinus fat invagination refers to the protrusion of tumor tissue into the renal sinus and its direct contact with the matrix or fat of the renal sinus, which is considered a manifestation of renal sinus invasion.^[2]^

Although most MESTKs are benign tumors, few studies have addressed their growth rate. We found 1 report of a case of MESTK that showed a 2 cm increase in size in 6 weeks.^[14]^ The case 1 in our study, the differences in the measurements from multiple preoperative examinations over 22 months may have been caused by different image levels of measurement (Fig. 1). However, the size of the tumor did not change markedly, and it showed a slow growth rate. The lesions were mainly multilocular cystic from the time of diagnosis to the time of surgery. Imaging tests showed no obvious septation thickening and no significant increase in solid components. However, malignant transformation of tumor components into carcinoma, sarcoma, carcinosarcoma, and malignant rhabdomyoma has been reported in the literature, indicating the malignant potential of MESTK.^[12,17]^ The best treatment for MESTK is surgery. The prognosis of benign MESTK after partial or radical nephrectomy is good, and recurrence or metastasis is rare. However, malignant MESTK can show recurrence, lymph node metastasis, and venous cancer thrombus, among other complications.^[12]^

MESTK is a rare clinical finding and preoperative imaging is difficult to diagnose. MESTK, which is predominantly cystic, should be distinguished from the following lesions: multilocular cystic nephroma (MCN), which presents as multilocular cystic with thin and smooth cyst walls and septa, without wall nodules and solid components, which are clearly demarcated from the surrounding normal renal tissue, and the degree of enhancement is lower than that of the surrounding renal parenchyma. According to the WHO classification, adult multilocular cystic nephroma are histologically classified as MESTK, and a differential diagnosis based on imaging is difficult.^[18,19]^ Cystic renal cell carcinoma is characterized by a cyst wall of uneven thickness with nodules, necrotic solid masses, and cystic degeneration; the contrast enhancement pattern is “fast in and fast out” type.^[8,13]^ MESTK, which is predominantly solid, should be distinguished from angiomyolipoma (AML) with epithelial cysts. AML is a common benign tumor of the kidney and angiomyolipoma with epithelial cysts is a cystic change in AML. AMLs contain considerable amounts of adipose tissue, and although some MESTK also contain adipose tissue, the vascular structure in renal AML is distinguished by marked contrast enhancement. Renal AML can occur in both men and women and is prone to hemorrhage, whereas MESTK is more common in perimenopausal women and hemorrhage is rare.^[3,16]^

In summary, MESTK is a clinically rare tumor commonly found in perimenopausal women with mild clinical signs. The tumors are mainly cystic, may contain more solid components, and the blood supply is not rich. Most lesions are benign, although some show a tendency to undergo malignant transformation. Most benign cystic lesions show a slow growth rate, although a few grow rapidly,and surgery is the best treatment. The biological behavior of MESTK is valuable in determining clinical treatment decisions.

As MESTK is a rare tumor, the main limitation of this report is the small number of cases, so the description of MESTK imaging and clinical manifestations may not be complete. We will continue to pay attention to MESTK.

## Acknowledgments

The authors would like to express their sincere gratitude to Jian-zhu Yang from the Department of Pathology, The First Hospital of Hebei Medical University. He carefully interpreted the patient’s pathological findings, which was of great help to this research. The achievements of this research would not have been possible without his expertise and skills and his hard work and contributions.

## Author contributions

**Formal analysis:** Ning Wang.

**Funding acquisition:** Ning Wang.

**Investigation:** Ning Wang, Shuo Wang, Xiang-Yang Chang, Bao-Gen Zhao.

**Methodology:** Li Zhang, Yong Wang.

**Supervision:** Li Zhang.

**Writing – original draft:** Ning Wang, Li Zhang.

**Writing – review & editing:** Ning Wang, Li Zhang, Yong Wang.
